# Pandan Lipophilic Bioactives Bind Milk Proteins with Gastric-Intestinal Stage-Specific Antioxidant Activity

**DOI:** 10.3390/antiox15101231

**Published:** 2026-09-23

**Authors:** Guanhua Lou, Shuzhen He, Zhen Feng, Xiaowei Qin, Haode Chang, Junyi Zhang, Wei Cheng, Fei Liu, Chunhe Gu

**Affiliations:** 1 Key Laboratory of Dairy Science, Ministry of Education, College of Food Science, Northeast Agricultural University, 600 Changjiang Road, Harbin 150030, China; arose12@126.com (G.L.); jyzhang9591@163.com (J.Z.); 19984078492@163.com (W.C.); 2 Spice and Beverage Research Institute, Chinese Academy of Tropical Agricultural Sciences, Wanning 571533, China; heshuzhen@catas.cn (S.H.); fengzhen@catas.cn (Z.F.); qinxiaowei@catas.cn (X.Q.); naachan98@outlook.com (H.C.)

**Keywords:** pandan, lipophilic bioactive compounds, milk protein, mechanism of interaction, antioxidant activity

## Abstract

Pandan (*Pandanus amaryllifolius*), a tropical aromatic plant, is a rich source of lipophilic bioactive compounds, notably characterized by high levels of squalene, phytol, and β-sitosterol, which exhibit antioxidant and lipid-lowering effects. Bovine milk proteins (α-casein, β-casein, and β-lactoglobulin) are widely used as delivery matrices owing to their nutritional value and technological functionality. In this study, molecular docking, fluorescence spectroscopy, circular dichroism spectroscopy, Fourier-transform infrared spectroscopy, and an in vitro digestion model were employed to systematically investigate the interactions between the major lipophilic bioactive compounds from pandan and bovine milk proteins. The results demonstrated that these compounds bound to milk proteins through hydrophobic interactions and van der Waals forces, resulting in the formation of protein–ligand complexes. Such interactions induced distinct conformational changes in the proteins, with caseins adopting a more compact structure, whereas β-lactoglobulin underwent localized unfolding. Furthermore, in vitro digestion revealed that the protein–bioactive systems exhibited distinct digestion-stage-dependent DPPH• and ABTS•+ radical-scavenging capacities, with the complexes displaying phase-dependent radical-scavenging responses during both gastric and intestinal phases. These findings provide molecular-level insights into the interactions between pandan bioactives and milk proteins and provide a theoretical basis for developing functional dairy products enriched with pandan-derived ingredients.

## 1. Introduction

Pandan (*Pandanus amaryllifolius*), a tropical aromatic plant, is widely used in Southeast Asia as a natural flavoring and coloring agent, and its unique aroma and nutritional value have attracted significant attention. Pandan leaves contain a wide range of lipophilic bioactive compounds, among which squalene, phytol, and β-sitosterol are the most abundant and frequently reported representatives. These compounds belong to distinct chemical classes: squalene (a triterpene), phytol (a diterpene alcohol), and β-sitosterol (a phytosterol) [1,2]. Therefore, these three compounds were selected as representative lipophilic bioactives of pandan. All three possess notable biological activities and substantial potential for functional food applications. However, their practical application is limited by poor water solubility, low bioavailability, phytothermal instability, and susceptibility to oxidative degradation during gastrointestinal digestion [1,3,4]. Consequently, plant-derived lipophilic bioactives have attracted growing interest in functional food development [5].

These compounds have been extensively studied for their antioxidant capacity, particularly free radical scavenging and oxidative stress alleviation [6]. However, the application of most lipophilic bioactive compounds in food systems remains limited because of their poor water solubility, susceptibility to light and heat, and limited dispersibility in aqueous food systems [7]. To address these limitations, protein-based delivery systems have emerged as a promising strategy. Through non-covalent interactions, proteins can bind lipophilic bioactive compounds, including squalene, phytol, and β-sitosterol, thereby altering their physicochemical behavior and dispersion characteristics [8,9]. Among these proteins, milk proteins, particularly casein micelles, have attracted considerable attention as natural delivery systems because of their unique porous structure and hydrophobic domains, which enable them to bind lipophilic bioactive compounds, improve their dispersion in aqueous environments and influence their physicochemical behavior and release during digestion [10].

Accordingly, this study aimed to characterize the interactions of squalene, phytol, and β-sitosterol with major bovine milk proteins; compare the radical-scavenging capacities of the individual proteins, individual bioactive compounds, and their corresponding complexes before digestion; and further evaluate the stage-dependent changes in DPPH• and ABTS•+ radical-scavenging responses of these protein–bioactive systems during simulated gastrointestinal digestion. The study focuses on molecular interactions, the potential contributions of the individual components to the initial radical-scavenging capacities, and digestion-stage-dependent changes.

## 2. Materials and Methods

### 2.1. Chemical Reagents

Squalene (sq, purity ≥ 98%), phytol (phy, purity ≥ 90%), β-sitosterol (β-sito, purity ≥ 98%), α-casein (α-CN, purity ≥ 70%), β-casein (β-CN, purity ≥ 98%), β-lactoglobulin (β-LG, purity ≥ 90%), reagents used in the in vitro digestion model (including α-amylase, pancreatin, pepsin, and bile salts), and all HPLC-grade solvents and reagents (acetonitrile, formic acid) were purchased from Shanghai Yuanye Bio-Technology Co., Ltd. (Shanghai, China). Unless otherwise specified, all other chemicals and reagents were of analytical grade.

### 2.2. Preparation of Stock Solutions of Major Milk Proteins and Bioactive Compounds

The α-CN, β-CN, and β-LG powders were dissolved in 0.1 mM PBS buffer (pH 7.4) to obtain protein solutions with a final concentration of 50 μM. The protein solutions were stored in amber centrifuge tubes at 4 °C and diluted with 0.1 mM PBS buffer (pH 7.4) before use. Sq, phy, and β-sito were dissolved in anhydrous ethanol to prepare 10 mM stock solutions, stored in amber centrifuge tubes at −20 °C, and diluted with anhydrous ethanol prior to use. Compared with the milk proteins, these compounds were treated as low-molecular-weight bioactive ligands in the present study.

### 2.3. Molecular Docking Research

The predicted three-dimensional (3D) structures of α-CN (P02662), β-CN (P02666), and β-LG (P02754) were obtained from the UniProt database. The structures of sq, phy, and β-sito were downloaded from the PubChem database. Molecular docking was performed using AutoDock 4.2 together with AutoDock Tools 1.5.7, and the docking results were visualized using Discovery Studio Visualizer [11].

### 2.4. Fluorescence Spectroscopy

Fluorescence experiments were conducted using an F-7000 fluorescence spectrometer (Hitachi, Tokyo, Japan) equipped with a 10 mm quartz cuvette at 298.15 K, following a previously reported method with slight modifications [12]. To minimize fluorescence interference from tyrosine residues in proteins, tryptophan was selectively excited by setting the excitation wavelength at 295 nm, the emission wavelength range to 290–500 nm, the scan rate to 1200 nm/min, and both the excitation and emission slit widths to 2.5 nm. The three proteins were added separately in PBS buffer (pH 7.4) and allowed to dissolve overnight at 4 °C to obtain 4 μM protein stock solutions.

In the fluorescence titration experiment, the ligand stock solution was added dropwise to 4 mL of protein solution (4 μL per addition) for a total of 10 additions, resulting in a final ligand concentration of 40 μM. The total ethanol concentration remained below 1% (*v*/*v*), which did not cause protein denaturation. After each addition, the mixture was thoroughly stirred and allowed to stand for 3–5 min to reach equilibrium, after which the fluorescence spectrum was recorded.

### 2.5. Circular Dichroism Studies (CDs)

CD measurements were performed on a Chirascan V100 circular dichroism spectrometer (Applied Photophysics Limited, Leatherhead, Surrey, UK) at 298.15 K under continuous nitrogen purging using a 1 mm path-length quartz cuvette. Following a previously reported method with slight modifications [13], scans were performed over the wavelength range of 190–260 nm, with the spectral resolution, scan rate, response time, and slit width set to 0.2 nm, 100 nm/min, 2 s, and 1 nm, respectively. CDNN software version 2.1 was used to analyze the CD spectra and estimate the secondary structure composition of the proteins after complex formation. The protein concentration was 2 μM, and the molar ratio of protein to small molecule was 1:2.

### 2.6. Fourier-Transform Infrared Spectroscopy (FTIR)

A Nicolet 6700 FTIR spectrometer (Thermo Fisher Scientific, Waltham, MA, USA) was used to analyze the interactions between proteins and lipophilic bioactive compounds. The protein concentration was 1 μM, and the molar ratio of protein to lipophilic bioactive compounds was 1:2. Preliminary experiments confirmed that the binding reached saturation at this molar ratio. Infrared spectra of the mixtures were obtained using the freeze-drying method. Each freeze-dried sample powder was mixed with potassium bromide in a 1:100 ratio and compressed into pellets. Spectra were recorded with a resolution of 4 cm^−1^ over a wavenumber range of 4000–400 cm^−1^, and the resulting spectra were subjected to baseline correction and normalization prior to further analysis. The spectral region of 1700–1600 cm^−1^ (amide I region) was selected to analyze changes in the secondary structure of protein–bioactive compound complexes. Spectral resolution was enhanced using second-derivative and Fourier self-deconvolution methods; peak fitting was performed using Gaussian functions; and the relative contents of secondary structures—α-helices, β-sheets, β-turns, and random coils—were calculated using PeakFit Version 4.12 software [14].

### 2.7. In Vitro Digestion

The in vitro digestion experiment was conducted in accordance with the INFOGEST in vitro digestion method, with appropriate modifications [15,16]. The digestion process consisted of three stages: oral, gastric, and intestinal. First, the sample (with a molar ratio of protein to lipophilic bioactive compounds of 1:2) was mixed with simulated saliva in a 1:2 (*v*/*v*) ratio. The pH of the system was adjusted to 7.0, and the mixture was incubated at 37 °C for 3 min. Simulated gastric juice was then added to the composite system, along with pepsin, to achieve a final enzyme activity of 2000 U/mL. The pH was adjusted to 3.0 using 1 M hydrochloric acid, and the mixture was shaken and digested for 2 h at 37 °C. Upon completion of gastric digestion, simulated intestinal fluid was added, along with trypsin and bile salts, to achieve a final trypsin concentration of 100 U/mL and a final bile salt concentration of 10 mM. The pH was adjusted to 7.0 using 1 M NaOH, and the mixture was digested for 2 h at 37 °C.

After the gastric and intestinal digestion were completed, the digested samples were collected separately, and ethanol containing 0.1% formic acid was added to terminate enzymatic activity. The digested mixtures were then filtered through 30–50 μm filter paper for subsequent determination of the antioxidant activity of the in vitro digesta.

### 2.8. Determination of DPPH• and ABTS•+ Radical-Scavenging Capacities of Milk Proteins, Bioactive Compounds, and Their Complexes Before and During In Vitro Digestion

This study employed DPPH and ABTS radical-scavenging assays to systematically analyze the antioxidant capacity of samples at different stages of in vitro digestion. In addition to the digested protein–bioactive complexes, the radical-scavenging capacities of the individual milk proteins (α-CN, β-CN, and β-LG), individual bioactive compounds (β-sitosterol, phytol, and squalene), and their corresponding complexes before digestion were determined separately. The individual proteins and bioactive compounds were tested at the same final concentrations as those present in the corresponding complexes.

The DPPH radical-scavenging activity was determined according to the method described by Munteanu and Apetrei [17], with minor modifications. For each sample group, 150 μL of the sample solution was mixed with 150 μL of a 0.159 mM DPPH solution prepared in ethanol. After incubation in the dark for 30 min, the absorbance was measured at 517 nm. The results were expressed as μg Trolox/mL based on the Trolox standard curve (R^2^ = 0.9992). The determination of ABTS radical-scavenging activity was performed according to the method described by Munteanu and Apetrei [17], with slight modifications. ABTS stock solution was mixed with K_2_S_2_O_8_ and incubated in the dark at 4 °C for 12 h to generate the ABTS^+^ radical cation. The resulting solution was then diluted with absolute ethanol to an absorbance of 0.70 ± 0.10 at 734 nm. For each sample group, 10 μL of the sample solution was mixed with 190 μL of the ABTS working solution; after incubation in the dark for 6 min, the absorbance was measured at 734 nm. The results were expressed as μg Trolox/mL based on the Trolox standard curve (R^2^ = 0.9971).

### 2.9. Statistical Analysis

All experiments were conducted in triplicate, and the results were expressed as the mean ± standard deviation (SD). Statistical analysis was performed using IBM SPSS Statistics 27.0. One-way analysis of variance (ANOVA) was used to compare overall differences, followed by Tukey’s test to assess the significance of differences between groups. A value of *p* < 0.05 was considered statistically significant.

During manuscript preparation, generative AI was used solely for English language editing and rewording, not for data generation, analysis, or interpretation. The authors reviewed all the text and assume full responsibility for the publication’s content.

### 2.10. Experimental Workflow

A schematic overview of the overall experimental workflow is presented in Figure 1.

## 3. Results and Discussion

### 3.1. Interactions Between Major Milk Proteins and the Lipophilic Bioactive Components in Pandan

#### 3.1.1. Molecular Docking Research

Figure 2 shows the molecular docking models of α-CN, β-CN, and β-LG with lipophilic bioactive compounds. Table 1 lists the amino acid residues involved in the interactions between the proteins and the lipophilic bioactive compounds, the number of hydrogen bonds, and the binding energies.

In the milk protein systems, α-CN, β-CN, and β-LG exhibit distinctly different conformational characteristics, which directly determine their steric hindrance and thermodynamic behavior encountered in binding to small-molecule ligands. α-CN and β-CN are typical intrinsically disordered proteins. They are rich in proline, lack a compact, regular fold, and adopt highly flexible extended or random coil conformations [18]. In particular, β-CN possesses strong amphiphilicity and a flexible polypeptide chain, enabling it to bind to ligands through conformational adaptation; however, this binding is often accompanied by significant changes in conformational entropy [19].

In contrast, β-LG is a highly ordered globular whey protein belonging to the lipocalin family. Its tertiary structure consists of eight antiparallel β-sheets that form a characteristic central hydrophobic β-barrel, together with an external α-helix. This rigid hydrophobic cavity provides a low-polarity microenvironment for small molecules and serves as the primary binding site for lipophilic ligands [20]. The flexible conformations of α-CN and β-CN, together with the rigid hydrophobic cavity of β-LG, form the structural basis for the distinct ligand-binding affinities and binding entropy losses of these proteins.

Molecular docking results indicate that all three lipophilic bioactive compounds (sq, phy, and β-sito) can spontaneously bind to α-CN, β-CN, and β-LG (binding free energy, ΔG < 0). Among these ligands, β-sito exhibited the highest binding affinity, with binding energies of −4.59 kcal/mol for α-CN, −3.84 kcal/mol for β-CN, and −4.89 kcal/mol for β-LG. This high binding affinity may be attributed to its rigid four-ring sterol structure, which allows tighter interactions with the hydrophobic regions of the proteins [6]. In contrast, phytol possesses 14 rotatable bonds and exhibits extremely high molecular flexibility. The torsional entropy loss incurred during binding is substantial, resulting in relatively lower binding affinity toward the proteins. Squalene, with its flexible structure featuring a main chain of up to 24 carbon atoms, must incur a considerable torsional entropy penalty when entering the protein binding pocket [21]. These findings demonstrate that, for lipophilic molecules, hydrophobicity alone does not necessarily confer the highest binding affinity; molecular rigidity and the hydrogen-bonding capacity of polar groups play critical roles in carrier-mediated transport by milk proteins.

In the α-CN and β-CN systems, the binding of all three lipophilic bioactive compounds was primarily driven by hydrophobic interactions and van der Waals forces, with no significant hydrogen bonding detected. In contrast, owing to the compact binding cavity of β-LG, both phytol and β-sito formed hydrogen bonds. Specifically, β-sito formed two hydrogen bonds with Asn109, whereas phytol formed one hydrogen bond with Glu134. Therefore, the binding of these compounds to β-LG was driven by hydrophobic interactions and van der Waals forces, with hydrogen bonds additionally contributing to the binding of phytol and β-sito.

Based on the molecular docking results, β-LG exhibited greater potential as a carrier for lipophilic ligands than α-CN and β-CN.

#### 3.1.2. Fluorescence Spectra

The aromatic amino acid residues present within protein molecules, particularly tryptophan and tyrosine, confer intrinsic fluorescence properties, providing direct spectroscopic evidence for elucidating protein–ligand interaction mechanisms [22]. To effectively minimize fluorescence interference from tyrosine and simplify spectral analysis, the excitation wavelength was set to 295 nm. At this wavelength, tyrosine exhibits negligible absorption; therefore, the measured intrinsic fluorescence signal is predominantly attributed to the Trp residue. This single-Trp probe is widely used to monitor protein–ligand interactions and changes in the polarity of the local microenvironment [23].

The number and location of tryptophan residues vary among different milk proteins, and these differences cause them to exhibit distinct fluorescence responses upon binding lipophilic ligands. For example, α-CN consists primarily of two subtypes, αS1-CN and αS2-CN, each containing two tryptophan residues (Trp-164/199 and Trp-109/193, respectively) [24,25]. In contrast, β-CN, an intrinsically disordered protein, contains only a single tryptophan residue (Trp-143) and can therefore serve as a specific single-probe model [26,27]. Although β-LG contains four tyrosine residues (Tyr-20, Tyr-42, Tyr-99, and Tyr-102), selective excitation at 295 nm eliminates fluorescence interference from tyrosine residues, allowing the spectral response to primarily reflect changes in the microenvironment of its two tryptophan residues (Trp-19, deeply buried within a hydrophobic cavity, and Trp-61, located on the protein surface) [28]. Changes in the polarity of the microenvironment surrounding these tryptophan residues can lead to fluorescence quenching or shifts in the emission maximum, thereby providing a spectroscopic basis for characterizing protein conformational changes and their specific interactions with lipophilic ligands.

Tryptophan residues within proteins are extremely sensitive to the polarity of their local microenvironment. At an excitation wavelength of 295 nm, the intrinsic fluorescence emission peak of bovine milk proteins is observed between approximately 330 and 340 nm. When small-molecule ligands interact with the proteins, this often results in a reduction in the Trp fluorescence signal, a phenomenon known as fluorescence quenching [29].

Figure 3 shows the fluorescence emission spectra of the three proteins with lipophilic bioactive compounds. Not all lipophilic bioactive compounds exhibited detectable binding to milk proteins; the experimental results demonstrate strong structural selectivity. Among all tested combinations, significant fluorescence quenching was observed in the α-CN-phy, β-CN-phy, β-LG-phy, and β-LG-sito groups. Table 2 presents the fluorescence values of the protein–ligand mixtures (F_mix_) and the background fluorescence of the free ligands (F_lig_) at 332–334 nm. The true protein fluorescence value (F_true_) was calculated as F_mix_ − F_lig_. As the concentration of phy increased, the intrinsic fluorescence intensities of α-CN, β-CN, and β-LG all exhibited a systematic, concentration-dependent decrease. The addition of β-sito to β-LG also induced significant fluorescence quenching. This systematic decrease in fluorescence intensity indicates that these specific ligands accessed the internal or surface hydrophobic regions of the proteins and interacted with Trp residues.

It is worth noting that no significant fluorescence quenching was observed in the remaining experimental groups, including the combinations of squalene with all three milk proteins and β-sitosterol with the two caseins (α-CN and β-CN). This difference—some ligands caused fluorescence quenching while others did not—indicates that only phytol exhibited detectable interactions with all three proteins, whereas the rigid and bulky β-sitosterol appeared to preferentially bind within the β-barrel cavity of β-LG. In contrast, squalene may undergo self-aggregation because of its high hydrophobicity, thereby reducing its interaction with milk proteins.

Furthermore, as the ligand concentration increased, none of the four groups of complexes exhibited a significant shift in the emission maximum wavelengths. This phenomenon suggests that, although ligand binding masks the Trp emission, it does not cause severe denaturation of the protein’s tertiary structure, nor does it expose the Trp to a more polar aqueous environment. This indicates that the lipophilic ligand undergoes static quenching by forming a stable ground-state complex with the bovine milk protein [30].

Meanwhile, the molecular docking results discussed earlier indicate that phytol binds to α-CN and β-CN solely through hydrophobic interactions and van der Waals forces, without hydrogen bonding, but is instead surrounded by a hydrophobic network composed of numerous nonpolar amino acid residues. The strong hydrophobic properties of the long hydrocarbon chain of phytol drive its spontaneous entry into and occupancy of the hydrophobic region near the tryptophan residue. This occupation of the hydrophobic microenvironment by the ligand and its displacement of surrounding water molecules may represent an important molecular basis leading to the static quenching of intrinsic tryptophan fluorescence.

### 3.2. The Effect of Interactions on the Secondary Structure of Cow’s Milk Proteins

Protein secondary structure mainly comprises α-helices, β-sheets, β-turns, and random coils. These structures are mainly formed and stabilized by hydrogen bonds between the carbonyl (C=O) and amide (N–H) groups of the peptide backbone. In this study, the secondary structures of the proteins after interaction were characterized using FTIR and CD. The amide absorption bands in protein infrared spectra primarily include amide I, amide II, and amide III, among which the amide I band is the most sensitive to changes in protein secondary structure. The CD and FTIR spectra of proteins interacting with lipophilic bioactive compounds are shown in Figure 4. The percentages of the secondary structural components of the individual proteins and their corresponding protein–bioactive systems are summarized in Table 3.

α-CN and β-CN are typical intrinsically disordered proteins (IDPs) with a highly flexible structure and a lack of ordered folding. In Figure 4a,b, both free α-CN and β-CN exhibited distinct negative ellipticity bands near 200–210 nm, which are primarily attributed to a high proportion of random coil structure and a small amount of β-sheet structure in their conformation. Upon the addition of lipophilic bioactive compounds, the CD spectra of the casein underwent noticeable changes: in the α-CN system, the intensities of the negative ellipticity bands in the CD spectra all showed an increasing trend following the addition of sq, phy, and β-sito, with the most significant increase observed after the addition of phy. These results suggest that the binding of lipophilic ligands induced the rearrangement of the α-CN peptide chains. Ligands bind to the hydrophobic regions of the proteins via hydrophobic interactions, prompting structural changes in the proteins. In the β-CN system, the addition of ligands similarly caused a slight decrease in the intensity of the negative ellipticity bands (particularly in the sq group). Since β-CN contains a high proportion of hydrophobic amino acid residues, these lipophilic bioactive compounds readily associate with the nonpolar microenvironment of β-CN through hydrophobic interactions. The changes in CD signal intensity following binding reflect adaptive conformational fine-tuning of β-CN’s flexible backbone as it accommodates these hydrophobic small molecules.

Unlike casein, β-LG is a typical globular protein belonging to the lipocalin family. Its native structure consists primarily of antiparallel β-sheets forming a central hydrophobic cavity capable of binding various hydrophobic ligands. In Figure 4c, free β-LG exhibited a typical broad negative ellipticity band near 215 nm, which is a hallmark of a β-sheet-rich structure. Upon the addition of sq, phy, and β-sito, the shape of the β-LG CD spectrum remained largely unchanged, but the intensity of the negative ellipticity band near 215 nm decreased sharply. This significant reduction in signal intensity (particularly pronounced in the phy group) suggests partial disruption or rearrangement of the β-sheet structure, accompanied by changes in other secondary structural elements (such as random coils). This phenomenon is relatively common in studies of protein–lipophilic ligand interactions. Due to the strong hydrophobicity and large steric hindrance, these ligands—upon entering the hydrophobic cavity of β-LG or binding to its surface hydrophobic regions—through their intense hydrophobic interactions may partially disrupt the hydrogen-bonding network and van der Waals forces that maintain the native folded state of β-LG. To accommodate these lipophilic bioactive compounds, β-LG may undergo partial opening of its hydrophobic cavity, leading to localized loosening of the internal structure, a decrease in the rigidity of the protein backbone, and a shift in the overall conformation toward a more extended state.

In summary, the binding of all three lipophilic bioactive compounds can induce conformational changes in these three milk proteins, but the resulting effects vary depending on the protein’s native structure. For caseins (α-CN and β-CN), which lack well-defined structures, ligand binding tends to promote conformational rearrangement or fine-tuning, whereas for the globular protein β-LG, which possesses greater structural rigidity, the binding of lipophilic bioactive compounds may partially disrupt the native structural stability, leading to a loosening of the backbone and the loss of some secondary structures. These conformational changes further confirm the existence of strong noncovalent interactions between proteins and lipophilic bioactive compounds.

### 3.3. Radical-Scavenging Capacities of Individual Components and Protein–Bioactive Complexes Before and During In Vitro Digestion

Before simulated gastrointestinal digestion, the radical-scavenging capacities of the individual proteins, individual bioactive compounds, and their corresponding complexes were evaluated using both DPPH• and ABTS•+ assays (Table 4). The individual milk proteins exhibited negligible DPPH• radical-scavenging capacities, ranging from −1.21 to 0.82 μg Trolox equivalents/mL, but showed measurable ABTS•+ radical-scavenging capacities of 39.58~46.35 μg Trolox equivalents/mL. The individual lipophilic bioactive compounds showed DPPH• radical-scavenging capacities of 2.07~6.48 μg Trolox equivalents/mL and ABTS•+ radical-scavenging capacities of 19.18~25.25 μg Trolox equivalents/mL. Compared with the individual proteins, the bioactive compounds showed more evident DPPH• radical-scavenging responses, whereas the proteins exhibited higher ABTS•+ radical-scavenging capacities.

The protein–bioactive complexes exhibited DPPH• radical-scavenging capacities ranging from 2.26 to 8.39 μg Trolox equivalents/mL and ABTS•+ radical-scavenging capacities ranging from 59.02 to 78.94 μg Trolox equivalents/mL. Among the complexes, α-CN-β-sitosterol showed the highest DPPH• response, whereas β-LG-β-sitosterol exhibited the highest ABTS•+ capacity. The complexes generally showed higher apparent ABTS•+ capacities than either corresponding component alone, whereas the DPPH• responses depended on the specific protein–bioactive combination. These findings indicate that the bioactive compounds themselves may contribute to the radical-scavenging capacities measured for the corresponding complexes; therefore, the activities of the complexes cannot be attributed exclusively to protein–bioactive interactions. These results demonstrate that the radical-scavenging responses were assay-dependent and provide an initial reference for interpreting the changes observed during gastrointestinal digestion. However, the higher values observed for some complexes should not be interpreted as evidence of a synergistic effect, because additive or combined contributions from the protein and bioactive compound cannot be excluded.

Following the initial comparison of the individual components and undigested complexes, the effects of simulated gastrointestinal digestion on the radical-scavenging capacities of the protein–bioactive complexes were further evaluated (Figure 5). Overall, the DPPH• and ABTS•+ assays showed distinct digestion-stage-dependent responses. DPPH• radical-scavenging capacity reached its highest level during the gastric phase and subsequently decreased during the intestinal phase, whereas ABTS•+ radical-scavenging capacity remained relatively low during the gastric phase and increased markedly during the intestinal phase. These contrasting responses indicate that the two assays responded differently to digestion-induced changes in the protein–bioactive systems.

During simulated gastric digestion, acidic conditions and pepsin-mediated hydrolysis may modify protein conformation and the binding microenvironment of the associated lipophilic bioactive compounds. Because the response of the DPPH• assay in an ethanol-containing reaction system is influenced by the solubility, partitioning, and accessibility of hydrophobic radical-scavenging compounds, digestion-induced changes in compound exposure or partitioning may partly explain the increased DPPH• radical-scavenging capacity observed during the gastric phase [14]. By contrast, the relatively low ABTS•+ radical-scavenging capacity at this stage may reflect differences in the amount and composition of soluble peptides generated by peptic digestion compared with subsequent intestinal digestion [31]. Therefore, the divergent DPPH• and ABTS•+ responses may be associated with both assay chemistry and digestion-induced changes in the protein–bioactive systems.

During simulated intestinal digestion, further proteolysis and the addition of bile salts alter the physicochemical environment of the system. Redistribution of lipophilic bioactive compounds into bile-salt-associated colloidal or micellar structures may affect their accessibility in the DPPH• reaction medium, thereby partly contributing to the decreased DPPH• radical-scavenging capacity [32]. Meanwhile, intestinal proteolysis may generate soluble peptides containing electron- or hydrogen-donating amino acid residues. These peptides may be more readily dispersed in the aqueous phase and contribute to the increased ABTS•+ radical-scavenging capacity [33].

The assay-dependent trend observed during the intestinal phase is consistent with the findings of Jiménez-Monreal et al. [34], who reported that simulated gastrointestinal digestion of fruit and vegetable powdered smoothies fortified with whey protein concentrate (WPC80) decreased DPPH• radical-scavenging activity but enhanced ABTS•+ radical-scavenging activity. However, their multicomponent food matrix differed substantially from the purified milk protein–bioactive compound systems investigated in the present study. Jiang et al. [35] reported concentration-dependent synergistic radical-scavenging effects between chlorogenic acid and peptides in both whey protein and casein digests. Unlike chlorogenic acid, squalene, phytol, and β-sitosterol lack multiple phenolic hydroxyl groups and exhibit markedly different polarity and partitioning behaviors, which may partly account for the different magnitudes and digestion-stage-dependent responses observed in the present study. Because these studies differed in sample composition, concentration, digestion protocol, assay conditions, and reporting units, the comparisons were restricted to overall trends rather than absolute values. Moreover, because peptide profiles, individual bioactive-compound concentrations, and micellar partitioning were not directly determined, these proposed mechanisms should be regarded as tentative explanations.

Therefore, the radical-scavenging capacities of the digested protein–bioactive systems cannot be attributed exclusively to either protein-derived peptides or the bioactive compounds. Instead, the measured responses likely reflect their combined contributions, with the relative importance of each component depending on the digestion stage and the radical-scavenging assay used. Because peptide profiles and residual bioactive-compound concentrations were not directly quantified, their individual contributions could not be quantitatively distinguished.

DPPH• and ABTS•+ are synthetic radicals rather than physiologically relevant gastrointestinal oxidants; therefore, the results obtained from these assays should be interpreted as comparative chemical radical-scavenging capacities. Oxidative reactions during digestion may instead involve lipid hydroperoxides and secondary oxidation products generated from dietary lipids [36], as well as nitrite-derived reactive nitrogen species formed under acidic gastric conditions [37]. Accordingly, the physiological relevance of the observed radical-scavenging responses should be interpreted in the context of assay chemistry and the specific oxidative processes occurring during gastrointestinal digestion.

## 4. Conclusions

This study employed molecular docking, fluorescence spectroscopy, FTIR, circular dichroism (CD) spectroscopy, and an in vitro digestion model to investigate the interactions between bovine milk proteins and major lipophilic bioactive compounds from pandan, as well as the radical-scavenging capacities of the resulting protein–bioactive systems before and during in vitro digestion. The results showed that protein–ligand interactions were mainly associated with hydrophobic interactions and van der Waals forces. The predicted binding energies were negative (ΔG < 0), suggesting thermodynamically favorable binding. Phytol induced fluorescence quenching in all three milk proteins, whereas β-sitosterol produced pronounced fluorescence quenching in β-LG. The protein–ligand association was accompanied by distinct changes in protein secondary structure, including alterations in the proportions of α-helices, β-sheets, β-turns, and random coils. During simulated gastrointestinal digestion, the DPPH• and ABTS•+ radical-scavenging capacities of the protein–bioactive systems exhibited distinct digestion-stage-dependent responses. These variations may be associated with digestion-induced changes in protein structure, ligand partitioning, peptide generation, and the physicochemical environment created by bile salts. Overall, these findings provide molecular-level insights into the interactions between milk proteins and pandan-derived lipophilic bioactives and may support the future development of functional food formulations containing these compounds.

## Figures and Tables

**Figure 1:**
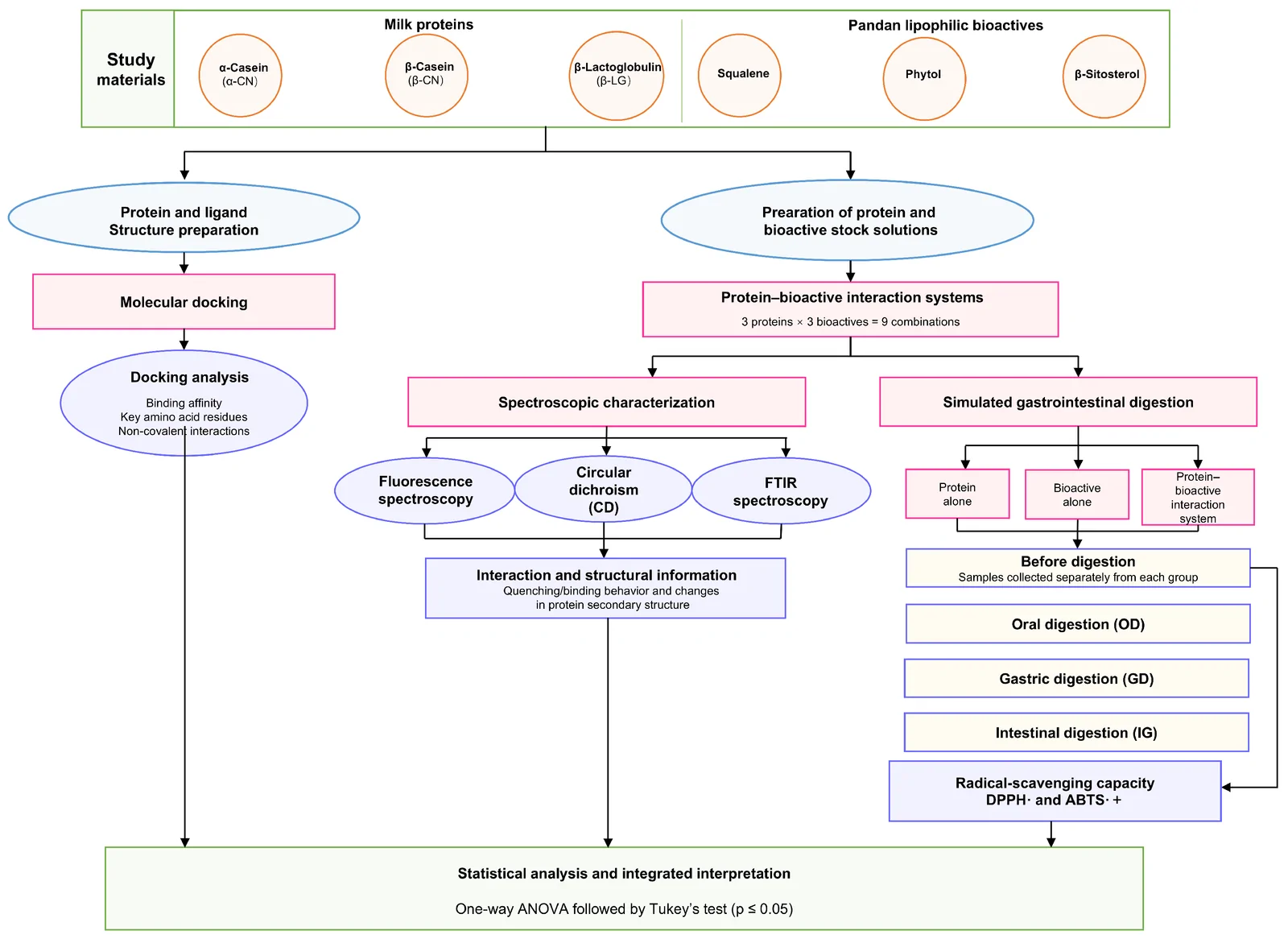
Schematic overview of the experimental workflow for investigating the interactions between major milk proteins and pandan-derived lipophilic bioactive compounds.

**Figure 2:**
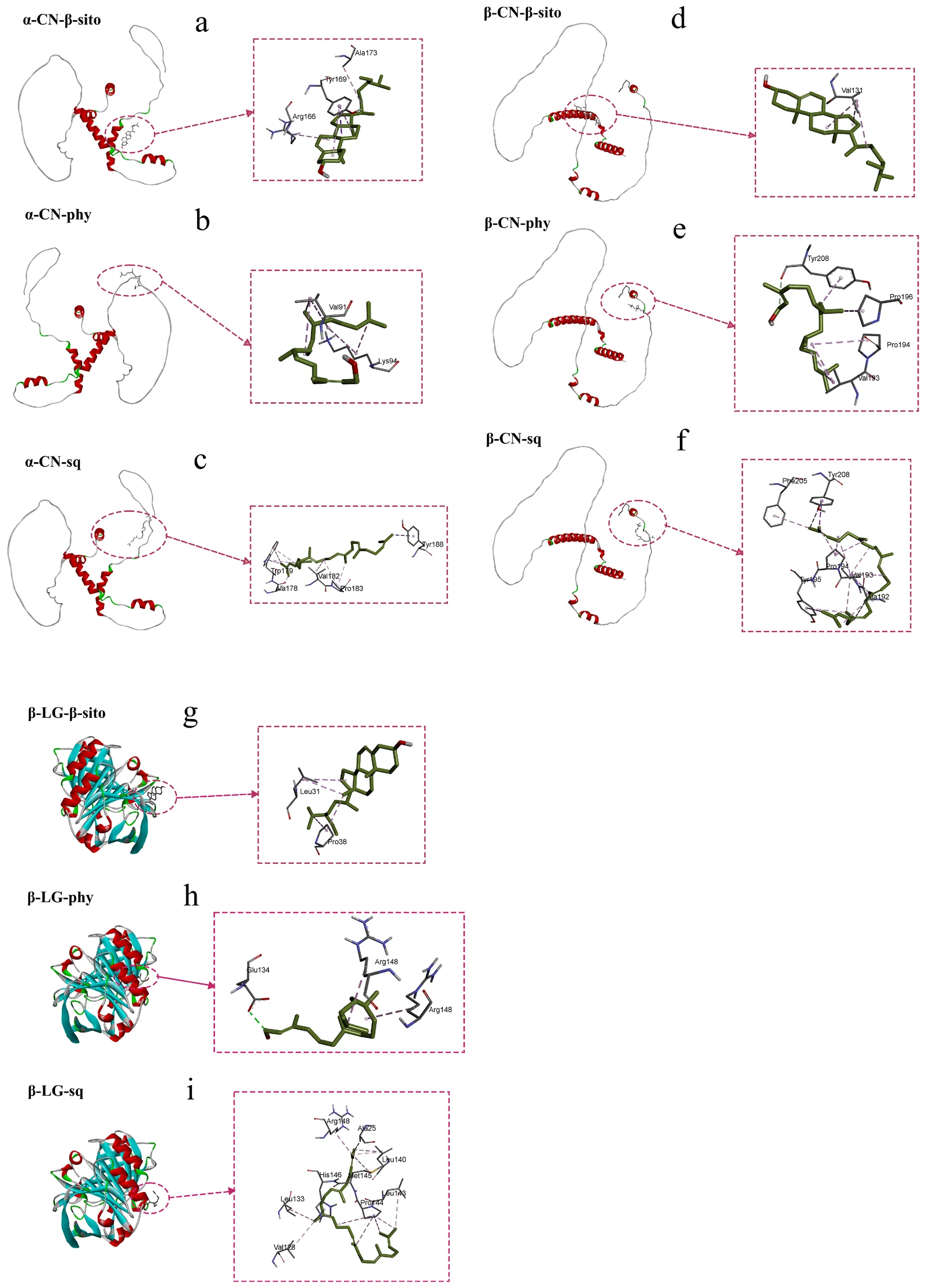
Molecular docking diagrams of α-CN, β-CN, β-LG, and lipophilic bioactive compounds. The diagrams show the binding sites of the proteins and small molecules, as well as the amino acid residues involved in the interactions. α-CN, α-casein; β-CN, β-casein; β-LG, β-lactoglobulin; sq, squalene; phy, phytol; β-sito, β-sitosterol. (**a**) α-Casein (α-CN)–β-sitosterol (β-sito); (**b**) α-CN–phytol (phy); (**c**) α-CN–squalene (sq); (**d**) β-casein (β-CN)–β-sito; (**e**) β-CN–phy; (**f**) β-CN–sq; (**g**) β-lactoglobulin (β-LG)–β-sito; (**h**) β-LG–phy; and (**i**) β-LG–sq.

**Figure 3:**
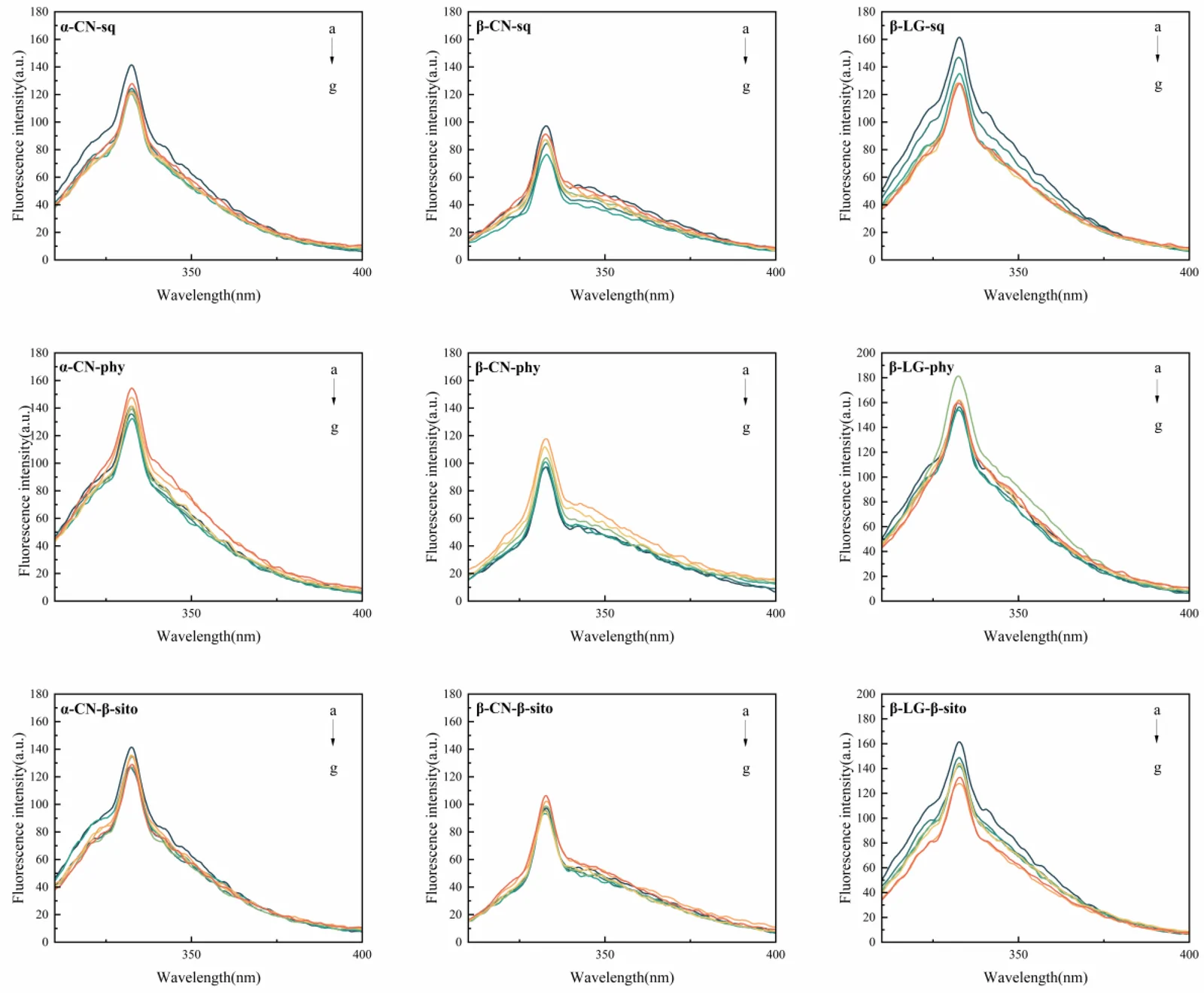
Fluorescence emission spectra of α-CN, β-CN, and β-LG in the presence of lipophilic bioactive compounds at a protein concentration of 4 × 10^−6^ mol/L. a → g: The concentrations of the lipophilic bioactive compounds are 0, 4 × 10^−6^ mol/L, 8 × 10^−6^ mol/L, 1.6 × 10^−5^ mol/L, 2.4 × 10^−5^ mol/L, 3.2 × 10^−5^ mol/L, and 4 × 10^−5^ mol/L, respectively. α-CN, α-casein; β-CN, β-casein; β-LG, β-lactoglobulin; sq, squalene; phy, phytol; β-sito, β-sitosterol.

**Figure 4:**
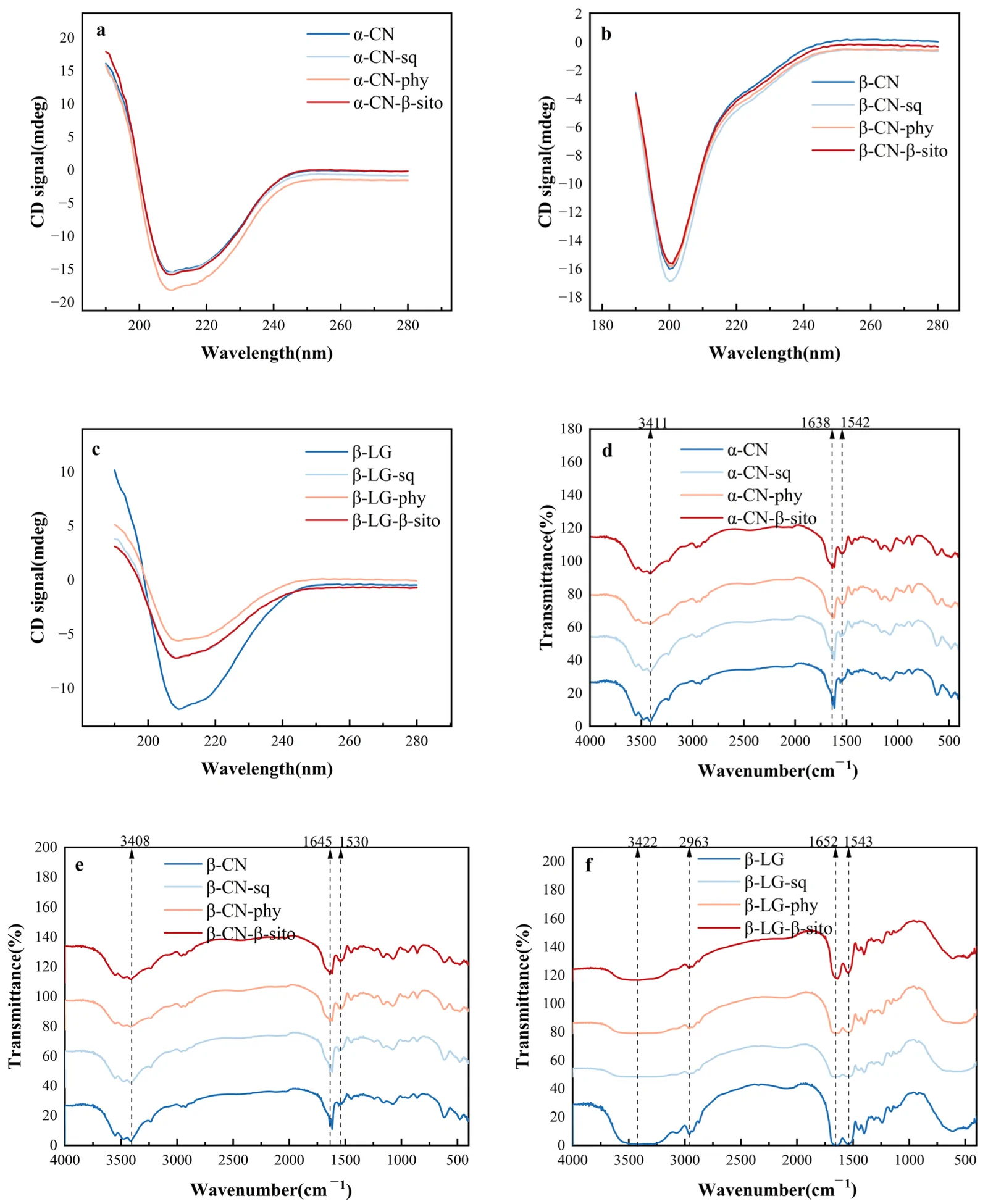
(**a**–**c**) The circular dichroism spectra of α-CN, β-CN, and β-LG after interaction with lipophilic bioactive compounds. The protein concentration was 2 × 10^−6^ mol/L, and the molar ratio of protein to lipophilic bioactive compounds was 1:2. (**d**–**f**) The Fourier-transform infrared (FTIR) spectra of α-CN, β-CN, and β-LG after interaction with lipophilic bioactive compounds. The protein concentration was 2 × 10^−6^ mol/L, and the molar ratio of protein to lipophilic bioactive compounds was 1:2. α-CN, α-casein; β-CN, β-casein; β-LG, β-lactoglobulin; sq, squalene; phy, phytol; β-sito, β-sitosterol.

**Figure 5:**
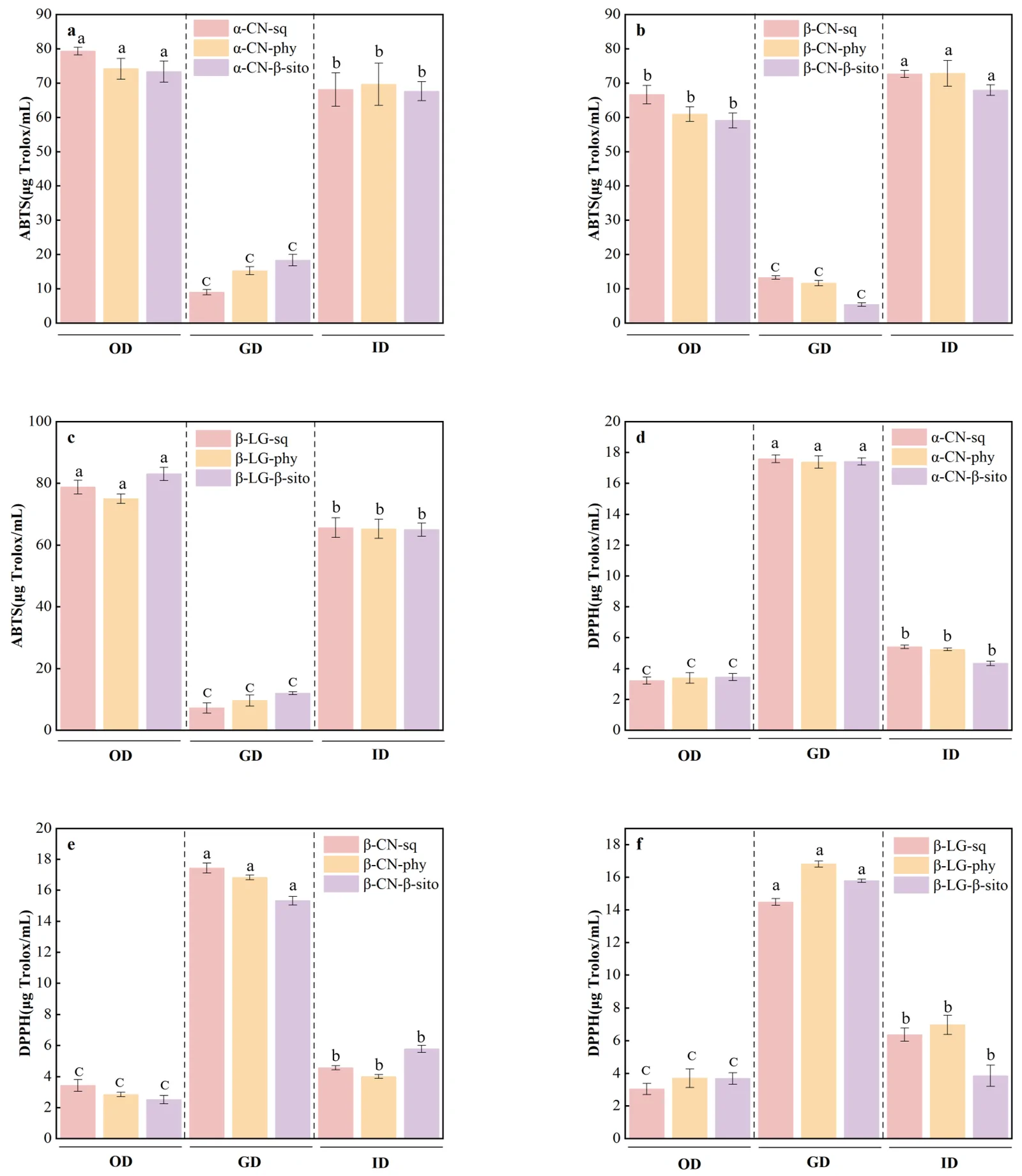
Antioxidant activity of digesta at different stages of in vitro simulated digestion. OD, GD, and ID represent the oral, gastric, and intestinal digestion stages, respectively. (**a**–**c**) The ABTS•+ radical-scavenging capacity (ABTS•+) of digesta after interaction between α-CN, β-CN, and β-LG and the three small molecules sq, phy, and β-sito, respectively; (**d**–**f**) the DPPH• radical-scavenging capacity (DPPH•) of digesta after interaction between α-CN, β-CN, and β-LG and the three small molecules sq, phy, and β-sito, respectively. Different lowercase letters above the bars indicate significant differences among digestion stages within the same protein–bioactive system (*p* ≤ 0.05), as determined by one-way ANOVA followed by Tukey’s test; bars sharing the same letter are not significantly different. The vertical dashed lines separate the OD, GD, and ID stages.

**Table 1:** The number of amino acid residues and hydrogen bonds involved in protein–lipid-soluble bioactive substance interactions and the free energy of binding at the optimal docking position.

Complex	Hydrophobic Interacting Residues	No. of H-Bonds	Binding Energy, kcal/mol
α-CN-sq	(<4.5 Å):ALA178, TRP179, TYR180, TYR181, VAL182, PRO183, GLY185, THR186, TYR18	0	−3.56
α-CN-phy	(<4.0 Å): LEU154, ILE157, VAL161, SER164, MET165, ALA168, PHE172	0	−1.16
α-CN-β-sito	(<3.5 Å) ASP172, TYR161, TYR174, PHE165	0	−4.59
β-CN-sq	(<3.5 Å) VAL193, ALA192, LYS191, GLN190	0	−2.36
β-CN-phy	(<3.5 Å) PRO194, PHE205, LYS191, VAL193	0	−0.75
β-CN-β-sito	(<3.5 Å) PHE134, GLU132, PRO130, TYR129	0	−3.84
β-LG-sq	(<3.0 Å) SER150, MET145, ASP137, ALA142, ARG148	0	−2.27
β-LG-phy	GLU134 (3.20 Å), (<3.5 Å) ASP137, ARG148, LEU140	1	−2.70
β-LG-β-sito	ASN109 (2.81 Å,3.23 Å), (<3.0 Å): ASN88, GLN115, ASN109, SER30	2	−4.89

**Table 2:** For the four systems exhibiting fluorescence quenching, F_mix_ represents the measured fluorescence intensity of the solution after interaction at 332–334 nm; F_lig_ represents the fluorescence intensity of the ligand mixed with PBS in the same proportion at 332–334 nm; and F_true_ = F_mix_ − F_lig_, which represents the protein’s intrinsic fluorescence intensity at 332–334 nm.

Mixed Systems	Molar Ratio (lig/pro)	F_mix_	F_lig_	F_true_
α-CN-phy	0	142.1	47.94	94.16
	2	133.3	54.39	78.91
	4	141.1	63.17	77.93
	6	142.8	72.62	70.18
	8	152.4	85.66	66.74
	10	155.5	90.43	65.07
β-CN-phy	0	98.01	47.94	50.07
	2	101.7	54.39	47.31
	4	105.1	63.17	41.93
	6	112.3	72.62	39.68
	8	125.1	85.66	39.44
	10	129.7	90.83	39.27
β-LG-phy	0	164.3	47.94	116.36
	2	156.5	54.39	102.11
	4	159.2	63.17	96.03
	6	163.3	72.62	90.68
	8	161.6	78.67	82.93
	10	162.5	90.43	72.07
β-LG-β-sito	0	164.3	47.94	116.36
	2	144.2	51.17	93.03
	4	147.7	57.03	90.67
	6	145	57.16	87.84
	8	128.7	54.62	74.08
	10	134.2	60.25	73.95

**Table 3:** Secondary structure percentages for proteins and protein–small molecule systems calculated using PeakFit Version 4.12 software. α-CN refers to α-casein, β-CN refers to β-casein, and β-LG refers to β-lactoglobulin. sq refers to squalene, phy refers to phytol, and β-sito refers to β-sitosterol.

Sample	β-Turn(%)	Random Coil(%)	α-Helix(%)	β-Sheet(%)
α-CN	23	36	0	40
α-CN-sq	21	37	0	41
α-CN-phy	28	26	0	46
α-CN-β-sito	29	26	0	44
β-CN	46	35	0	19
β-CN-sq	54	29	0	17
β-CN-phy	62	21	0	17
β-CN-β-sito	64	20	0	17
β-LG	49	10	16	24
β-LG-sq	54	8	21	17
β-LG-phy	62	8	13	17
β-LG-β-sito	64	6	14	16

**Table 4:** DPPH• and ABTS•+ radical-scavenging capacities of individual milk proteins, individual lipophilic bioactive compounds, and their complexes before in vitro digestion. Values are expressed as mean ± SD (n = 3). Different lowercase superscript letters within the same column indicate significant differences among samples (*p* < 0.05), as determined by one-way ANOVA followed by Tukey’s HSD test.

Sample	DPPH• (μg Trolox /mL)	ABTS•+ (μg Trolox/mL)
α-CN	0.003 ± 0.355 ^fg^	42.224 ± 1.162 ^ef^
β-CN	−1.211 ± 0.219 ^g^	39.578 ± 0.918 ^f^
β-LG	0.819 ± 0.282 ^efg^	46.350 ± 1.242 ^e^
β-sito	6.477 ± 0.552 ^ab^	22.419 ± 0.738 ^gh^
phy	6.282 ± 0.084 ^ab^	25.252 ± 0.682 ^g^
sq	2.070 ± 0.355 ^def^	19.184 ± 0.584 ^h^
α-CN–β-sito	8.387 ± 2.451 ^a^	73.756 ± 1.865 ^b^
α-CN–phy	5.221 ± 0.147 ^bc^	74.078 ± 1.605 ^b^
α-CN–sq	2.349 ± 0.524 ^de^	78.011 ± 2.086 ^ab^
β-CN–β-sito	3.779 ± 0.218 ^cd^	59.023 ± 1.453 ^d^
β-CN–phy	5.345 ± 0.329 ^bc^	61.104 ± 1.347 ^cd^
β-CN–sq	3.422 ± 0.244 ^cd^	65.407 ± 1.647 ^c^
β-LG–β-sito	3.357 ± 0.332 ^cd^	78.940 ± 1.844 ^a^
β-LG–phy	2.258 ± 0.241 ^de^	75.609 ± 1.735 ^ab^
β-LG–sq	4.519 ± 0.167 ^bc^	63.457 ± 2.178 ^cd^

## Data Availability

The original contributions presented in this study are included in the article. Further inquiries can be directed to the corresponding author.
